# Pragmatic trial design enhances diversity and retention among research participants with Lewy body diseases

**DOI:** 10.1038/s44400-026-00059-x

**Published:** 2026-02-02

**Authors:** Nghi Tran, Chen-Pin Wang, Sudha Seshadri, Pablo Coss, Okeanis E. Vaou, Leila Saadatpour, Carolyn Paiz, Diana Solis, Jessica Grimaldo, Sarah Erskine, Sarah Horn

**Affiliations:** 1 The University of Texas Health Science Center at San Antonio (UTHSCSA), San Antonio, TX USA; 2 The Glenn Biggs Institute for Alzheimer’s and Neurodegenerative Diseases, The University of Texas Health Science Center, San Antonio, TX USA

**Keywords:** Health care, Medical research, Neurology, Neuroscience, Psychology, Psychology

## Abstract

An ongoing pragmatic, randomized, unblinded clinical trial compares pimavanserin and quetiapine for psychosis in Lewy body diseases over 6 months (*n* = 60). Relative to local explanatory clinical trials, retention is higher (93% vs 40%, *p* < 0.001) and Hispanic enrollment greater (38% vs 21%, *p* = 0.043), reflecting the clinic demographics. Embedding pragmatic trials within routine care can enhance retention and diversity, thereby improving representativeness and real-world applicability of clinical research. Registry: ClinicalTrials.gov, TRN: NCT05590637; Registration Date: 18 October 2022.

Parkinson’s disease (PD) and dementia with Lewy bodies (DLB), collectively referred to as Lewy body diseases (LBDs), are neurodegenerative diseases that cause both abnormal movement such as tremor and bradykinesia as well as non-motor symptoms, including mood, cognitive, and behavioral problems. Psychosis - which manifests as hallucinations, illusions, delusions, or a false sense of presence or passage^1^ - is a particularly distressing non-motor symptom that worsens quality of life, heightens caregiver strain, and increases hospitalization, institutionalization, and mortality^2–6^.

Research efforts are underway to advance understanding and treatment options in LBDs, though the traditional, explanatory randomized controlled trials (RCTs) that are often used face considerable barriers to patient enrollment and long-term participation. In one survey of 832 individuals with PD, less than 10% had ever participated in a clinical trial, even though most respondents indicated they would be open to doing so^7^. Broader analyses show that about three quarters of investigators fall short of enrollment goals, and more than a quarter of studies close without enrolling any participants^8^. Maintaining participation after enrollment is also difficult, particularly when protocols require frequent visits and demanding procedures, which often depend on caregiver time and resources^9,10^. These barriers are even more pronounced in LBDs, where cognitive impairment complicates adherence, and caregivers carry a large share of the logistical responsibility. In addition, racial and ethnic minority groups continue to be strikingly underrepresented. Across decades of U.S.-based PD RCTs, fewer than 20% have reported participant race, and when they did, non-White individuals comprised only about 8% of enrollees despite representing roughly 20% of the older adult population^11^. More recent reviews show little progress, with over three-quarters of studies still lacking racial and ethnic data and minority enrollment remaining below 5%^12,13^.

Although explanatory RCTs remain central to establishing causality, their rigorous conditions can make results less applicable to routine clinical care and may overlook benefits meaningful to patients^14,15^. These studies emphasize internal validity through blinding, placebo use, narrow eligibility criteria, and extensive assessments. In real world settings, however, treatment response depends on more than the intervention itself. The clinician-patient relationship^16–18^, placebo effects^19,20^, and individual preferences^21,22^ all shape adherence and outcomes. In response, pragmatic clinical trial designs have been proposed as a way to study treatments under conditions that better reflect everyday medical practice^23^.

In this study, we describe a pragmatic randomized trial comparing two antipsychotic treatments, pimavanserin and quetiapine, for psychosis in LBDs over a period of six months. While pimavanserin is the only medication approved by the U.S. Food and Drug Administration specifically for PD psychosis, its real-world use is limited by cost, insurance restrictions, and concerns about its comparative effectiveness. Quetiapine is often prescribed as a more accessible and cost-effective alternative^24,25^. However, existing evidence for quetiapine use in LBDs is mixed, with studies limited by small sample sizes and inconsistent results, further illustrating limitations of explanatory RCTs^26–31^.

By integrating research into standard clinical workflows, this pragmatic trial aims to produce findings that are more applicable to real-world practice in a diverse population. To date, 60 participants (mean age 76 years) have enrolled (Table 1), comprising 43% of patients seen by the enrolling clinicians for this study (*n* = 141), and 25% of all patients (*n* = 239), with PD or DLB who were seen in the University of Texas Health Science Center at San Antonio (UTHSCSA) Neurology Clinic and prescribed quetiapine or pimavanserin during the study period, as identified using SlicerDicer (Epic Systems Corporation, Verona, WI). Thirty (50%) were randomized to receive pimavanserin and 30 (50%) to quetiapine. Of the total cohort, 52 (87%) are diagnosed with PD (including those with PD dementia (PDD)) and 8 (13%) with DLB. Twenty-four (40%) are women and 23 (38%) identify as Hispanic. At the time of this report, a total of 56 participants are enrolled and retained (93%), including 48 who have completed the study and 8 who remain active with a mean time since enrollment of 130 days. Four participants were lost to follow-up (2 due to death and 2 due to relocation or withdrawal). Among the 4 participants lost to follow-up, all are men and White, including 1 who identifies as Hispanic and 1 with unknown ethnicity. Based on ZIP codes, participants have a mean Social Vulnerability Metric (SVM) score of −0.41 (39.7 percentile), where negative SVM scores and lower SVM percentiles indicate less social vulnerability^32^.

**Table 1:** Baseline Characteristics of Enrolled Participants

Patient Characteristics	Total = 60
Age, mean ± SD (years)	76 ± 8
Sex, male, *n* (%)	36 (60)
Race, White, *n* (%)	56 (93)
Hispanic Ethnicity, *n* (%)	23 (38)
Social determinants of health	
Primary insurance, *n* (%)	
* Medicare/Medicare Advantage*	46 (77)
* Commercial (including managed care plans)*	12 (20)
* Tricare*	1 (2)
* Uninsured*	1 (2)
Marital status, *n* (%)	
* Married/domestic partnership*	41 (68)
* Single/separated/divorced*	11 (18)
* Widowed*	8 (13)
SVM metric, mean ± SD	-0.41 ± 1.29
SVM percentile, mean ± SD	39.70 (34.60)
Diagnosis, n (%)	
PD	52 (87)
* PD without dementia*	28 (54)
* PD with dementia (PDD)*^a^	24 (46)
DLB	8 (13)
Has DBS, n (%)	5 (8)
Baseline MDS-UPDRS Part III, mean ± SD (points)^b^	49 ± 14
H&Y, mean ± SD (points)^c^	3 ± 1
PD medication use, *n* (%)	
Carbidopa-Levodopa (any formulation)	53 (88)
Amantadine	10 (17)
Dopamine agonist	9 (15)
MAO-B inhibitor	7 (12)
COMT inhibitor	1 (2)
Adenosine A2A receptor antagonist	1 (2)
None	6 (10)
Cholinesterase inhibitor use, n (%)	22 (37)
Symptoms of psychosis, n (%)	
Hallucinations	58 (97)
Delusions	34 (57)
Treatment allocation, *n* (%)	
Pimavanserin	30 (50)
Quetiapine	30 (50)

*SD* standard deviation, *n* number, *SVM* Social Vulnerability Metric, *DBS* deep brain stimulator, *MDS-UPDRS* Movement Disorders Society Modified Unified Parkinson’s Disease Rating Scale, *H&Y* Hoehn and Yahr score, *MAO* Monoamine oxidase inhibitor, *COMT* Catechol-O-Methyltransferase.

^a^ Based on cognitive status documented in the medical record at the time of enrollment, or within 12 months after enrollment if no cognitive status documentation was available at enrollment.

^b^among 57 with data available.

^c^among 54 with data available.

The demographics of the trial cohort were compared both with the overall clinic population of people with PD (PwP) and with those who participated in explanatory RCTs at the trial center. Compared with the broader population of PwP followed in the UTHSCSA Movement Disorders Clinic, there are no significant differences in race, ethnicity, or sex among the trial participants (Table 2). When compared with participants enrolled in concurrent explanatory RCTs for people with PD and/or DLB within the Movement Disorders Division at our center (*n* = 68), this pragmatic trial enrolled a higher percentage of Hispanic participants (38% vs 21%; *p* = 0.043) and demonstrated improved retention (93% vs 40%; *p* < 0.001); there are no differences in sex or race.

**Table 2:** Comparison of the trial participant demographics to those of the overall PD clinic cohort

Patient Characteristics	Pragmatic trial participants, *n* = 60	Overall Clinic Cohort of PwP, *n* = 1111	*p*-value
Sex, male, *n* (%)	36 (60)	694 (62)	0.693
Ethnicity, *n* (%)			0.138
Hispanic	23 (38)	337 (30)	
Non-Hispanic	33 (55)	655 (60)	
Unknown	4 (7)	119 (11)	
Race *n* (%)			0.808^a^
White	56 (93)	922 (83)	
Black or African American	1 (2)	13 (1)	
Asian	1 (2)	11 (1)	
More than 1 race	1 (2)	7 (1)	
Unknown/Other	1 (2)	158 (14)	

^a^among patients with race data available.

This brief communication describes a pragmatic trial design for evaluating antipsychotic treatments in LBDs that proved both feasible and effective in achieving strong recruitment, high retention and greater inclusion of diverse participants. The retention rate and minority representation are higher than is typically reported in RCTs^11–13,33,34^, though similar patterns of high retention have been reported in other pragmatic PD trials. For example, in the PD MED study, 91% of the 1620 participants remained in the trial after one year and roughly 60% completed three-years of follow-up^35^.

The percentage of women enrolled (40%) is slightly below the estimated prevalence in PD (42–46%)^36,37^ but is at the higher end of the 30–40% range typically enrolled in RCTs^38,39^, and is consistent with our clinic demographics, suggesting that sex representation in this trial reflects that of PD patients in the underlying clinic population.

The racial, ethnic, and sex composition observed here likely results from two main factors: 1) the pragmatic design, which reduced common participation barriers; and 2) the demographic profile of our clinic population^10,40^. Table 3 summarizes our study’s degree of pragmatism using the PRECIS-2 framework^23^.

**Table 3:** Pragmatism of the trial design scored using the PRECIS-2 tool

PRECIS-2 Domain	Pragmatic Score range from 1 (very explanatory) to 5 (very pragmatic)	Description
Eligibility Criteria	5 (very pragmatic)	Anyone with PD or DLB psychosis likely to be a candidate for either medication in routine care
Recruitment	4 (rather pragmatic)	Usual appointments at diverse clinics, some advertising used
Setting	3 (equally pragmatic and explanatory)	Local academic center, multiple clinics, diverse socioeconomic backgrounds
Organization	4 (rather pragmatic)	No extra training for prescription management, 1:1 training for the trial protocol with the principal investigator, utilize extra research staff for record keeping and study management
Flexibility (Delivery)	5 (very pragmatic)	Medication prescribed per routine care at prescriber’s full discretion
Flexibility (Adherence)	5 (very pragmatic)	Medication managed per routine care
Follow-up	4 (rather pragmatic)	Mostly at routine appointments, some additional contact for outcome collection
Primary Outcome	4 (rather pragmatic)	NPI-Q not used in routine care, but is easily administered and targets psychosis severity
Primary Analysis	5 (very pragmatic)	Intention-to-treat using all available data

The broad eligibility criteria of our study enable nearly any patient with psychosis in PD or DLB to be considered for the trial, supporting more inclusive enrollment. Recruitment occurs during routine clinic visits with only limited outreach, keeping study activities closely aligned with usual care.

Financial costs, such as those incurred by travel, lodging, lost wages, or additional testing, have been recognized as major barriers to trial participation, particularly for patients from lower-income or underrepresented backgrounds^10,40^. In our study, assessments are scheduled during routine clinical 6-month follow-up visits, and data collection occurs alongside care with only minor extensions to visit length. This approach minimizes additional travel and time demands for patients and caregivers and thereby avoided burdens that often reduce retention^9,10^. Since both study arms use medications already prescribed in standard practice, no extra procedures or training were required for medication management, which also adds to the feasibility of the trial.

Language barriers are a known challenge to research participation^10^. At one major academic institution, a review of 1,492 studies showed that non-English speakers were ineligible to participate in almost half of the studies reviewed^41^. Our study sought to reduce this barrier by providing materials and consent forms in both English and Spanish, using bilingual staff, and arranging translators when necessary, allowing Spanish-speaking patients and families to participate fully.

The cohort’s mean SVM score is negative and the mean SVM percentile is lower than average, which indicates that participants on average are from less socially vulnerable areas^32^. However, the large standard deviation indicates a wide range of SVM scores among participants, with some living in higher-vulnerability communities. This supports the importance of maintaining accessibility features that lower participation burden across diverse socioeconomic backgrounds.

In addition, the outcome measures selected for this study reflect real-world considerations, including psychosis severity, medication adherence versus discontinuation, global impression of change, burden of neurology clinic care utilization, adverse events, and out of pocket treatment costs. Clinicians retain full discretion in treatment decisions, which adds to the practical relevance of results. Inclusion of caregiver burden as a key outcome recognizes the central role that families play when managing psychosis in LBDs. Together, these design elements align with proposed recommendations to address disparities in research^42^.

This study has several limitations. It is being conducted at a single academic center in a Hispanic majority city. While this helps improve representation among an often-underrepresented group, it may limit the generalizability of the results to other regions, healthcare settings, or demographic groups. Our study included only one Black participant, reflecting the clinic’s catchment population rather than differences in recruitment efforts. However, this highlights a broader challenge in neurology research, where Black patients remain consistently underrepresented. While pragmatic designs can lessen some logistical barriers, they cannot fully address structural inequities in access to specialty care or research participation. Future studies could strengthen partnerships with community organizations, expand outreach and trust-building, and ensure greater racial and cultural diversity among research and clinical teams to better address these inequities^10,42^. Centers with less diverse patient populations may consider collaborating with nearby institutions or community clinics to reach underrepresented groups in research.

The relatively modest sample size of the study reduces power for subgroup analyses and the ability to detect subtle differences between treatment groups. The open-label design introduces the possibility of bias, though this risk is mitigated by comparing two treatment arms receiving standard-of-care medications, without a placebo group. Treatment decisions, including dose adjustments and any changes in therapy, are made as part of routine care, which supports the pragmatic nature of the study but introduces variability that complicates precise comparisons between groups. Randomization stratification by center rather than by individual clinician may leave some residual influence from clinician practice patterns. Sensitivity analyses are planned to assess the extent of this effect.

Our findings support the feasibility of using a pragmatic trial design to evaluate antipsychotic treatments in LBDs. The study has achieved strong participant retention and successfully enrolled individuals from a historically underrepresented ethnic population. In doing so, it addresses pervasive challenges in neurodegenerative disease research regarding retention and representation. By reducing participation barriers and integrating research into routine care, pragmatic designs support more inclusive and patient-centered practices. Expanding such approaches in future studies may accelerate the application of evidence-based care, especially in underserved communities.

## Methods

### Study design and setting

This investigator-initiated, pragmatic, randomized, open-label clinical trial is being conducted at a single academic medical center in San Antonio, Texas. The trial compares the effectiveness and tolerability of quetiapine versus pimavanserin for the treatment of psychosis associated with Lewy body diseases, including PD and DLB. The study was registered on ClinicalTrials.gov, TRN: NCT05590637, Registration Date: 18 October 2022. This manuscript was prepared in accordance with the CONSORT 2025 statement and checklist (supplementary information)^43^.

The trial protocol was approved by the Institutional Review Board (IRB) at UTHSCSA (ID Number: 22-0198H) and is conducted in accordance with the Declaration of Helsinki and relevant regulatory requirements. Written informed consent is obtained from all participants or their legally authorized representatives prior to enrollment.

### Recruitment and enrollment

Participant recruitment began in 2022 and is paused for a planned interim analysis. Patients are identified through standard clinical workflows in neurology clinics. Eligibility screening and enrollment are performed during routine care encounters, and informed consent is obtained in person or remotely. No financial incentives are offered for participation. Both English- and Spanish-speaking patients are included. All study materials are available in both languages. Spanish-speaking participants are typically consented by bilingual clinicians or research staff, and certified interpreters are used when bilingual staff are unavailable, which is uncommon.

Eligible participants are adults with PD (including PDD) or DLB-related psychosis receiving care at the UTHSCSA or University Hospital Neurology Clinics (the latter is staffed by UTHSCSA Neurology faculty), for whom antipsychotic treatment is indicated. Diagnoses are made by movement disorders specialists during the course of routine clinical management. Clinical equipoise between quetiapine and pimavanserin must exist such that either medication is a reasonable treatment option, and the prescribing clinician must be comfortable prescribing and managing both. Patients meeting criteria are invited to participate by their clinician, who proceeds with enrollment or, if preferred by the clinician, involves a research coordinator to assist.

Exclusion criteria include any medical contraindication to either study medication, absence of a caregiver available to complete the Neuropsychiatric Inventory Questionnaire (NPI-Q)^44^, current use of an antipsychotic at the time of enrollment, or a prescribing clinician who is unwilling to manage both treatment options.

### Randomization and interventions

Participants are randomized in a 1:1 ratio to receive either quetiapine or pimavanserin. Seven clinicians participated in enrollment. Randomization is stratified by clinic site, performed using the NIH Clinical Trials Randomization Tool (https://ctrandomization.cancer.gov/tool/), and implemented using sequentially numbered, opaque, sealed envelopes. The allocated intervention is unblinded to all immediately after assignment. Consistent with the pragmatic design, clinicians retain full discretion in treatment decisions. All study medications are prescribed, managed, and adjusted as part of routine care by the treating clinicians, who do not have access to the random allocation sequence.

### Outcome measures

Outcomes are measured from baseline to six months (range: minus 2 weeks, plus 3 months). The primary outcome is the change in the combined NPI-Q hallucination and delusion item scores. Secondary outcomes include changes in the total NPI-Q score, clinician-rated Clinical Global Impression of Change (CGI-C), Patient Global Impression of Change (PGIC), Clinical Global Impression of Change for Caregiver Rating (CGI-C:CVR), time to discontinuation of the assigned medication, adverse events, mortality, change in the MDS-UPDRS Part III score^45^, and changes in other NPI-Q subdomains such as anxiety, agitation, and nighttime behaviors. A baseline formal cognitive assessment was not included, as study procedures were limited to measures that are quick and simple to obtain and most relevant to the study outcomes, in order to maintain pragmatism, minimize enrollment burden, and facilitate incorporation of study assessments into routine clinic visits. Planned exploratory outcomes include changes in the NPI-Q caregiver distress score, patient-reported out-of-pocket medication costs, and the frequency of patient contact with the neurology clinic, including visits, phone calls, and electronic medical record messages. Planned subgroup analyses include PD and DLB, Hispanic and non-Hispanic, and per-protocol treatment groups.

### Data collection and follow-up

Data are collected at baseline and again at the 6-month follow-up, which corresponds to a typical visit interval in our clinic, though some patients had additional visits during the trial period depending on clinical needs. In-person visits are preferred, though telemedicine visits are permitted. Data collected during visits include patient and caregiver interviews, medication records, and standardized assessments. Each visit is extended approximately five additional minutes to complete the consent process, randomization, and outcome measures. The NPI-Q is typically completed by the caregiver while other clinical activities, such as the neurological exam or prescription management, are underway. If caregivers are not physically present, the clinician or a research coordinator can collect the NPI-Q data by phone. Patients who do not return for an in-person visit during the follow-up period are contacted by phone to obtain outcome data. For phone or telemedicine follow-ups, the motor examination (MDS-UPDRS Part III) is not performed. Treating clinicians were individually trained by the principal investigator (PI) on the study protocol and assessments. A research coordinator trained in the study is available to assist upon request. The PI oversees data quality throughout the study. Some data are extracted from the electronic medical record (EMR) by the research team, including race and ethnicity, which are reported by participants and recorded in the EMR.

### Demographics comparison

The overall cohort of PwP included all patients seen in the UTHSCSA Neurology Clinic with an International Classification of Disease (ICD-10) code for PD. Electronic medical records were reviewed for PD diagnosis confirmation, and PwP were included in this cohort if they first saw a movement disorders specialist at UTHSCSA between 2014, when the Movement Disorders Clinic was established, and 2022, when the cohort was created.

The pragmatic trial cohort is compared with participants enrolled in concurrent explanatory RCTs for people with PD and/or DLB within the Movement Disorders Division at our center. Participants are considered retained if they enrolled in the study and have either completed the study or remain in the study, as opposed to those who screen failed, terminated the study early, or died.

### Statistical analysis

A prior retrospective comparison of quetiapine and pimavanserin estimated an effect size of 0.58 for psychosis outcomes^46^. Based on this estimate, a two-tailed α of 0.05, and 80% power, a total sample size of 94 participants (47 per arm) was calculated using a standard two-sample comparison of means. Because this power analysis was derived from retrospective data and therefore represents an imperfect estimate of the true effect size, an interim analysis has been planned for once 60 participants enrolled, including a repeat power calculation if the primary endpoint has not yet been reached.

Descriptive statistics were used to characterize the participants. Chi-squared tests were used to compare categorical variables between groups, except for comparison between trial participants with the overall clinic cohort, for which a chi-squared goodness-of-fit test was used.

Planned analyses for the trial outcomes include t-tests for continuous variables with normally distributed data, Wilcoxon rank-sum tests for non-normally distributed continuous data, and chi-squared tests for categorical variables, to compare characteristics between groups. Data will be analyzed on an intention to treat basis, with missing data omitted. Multivariable generalized linear models (or parametric lifetime model for medication discontinuation) with suitable outcome distributions will be used to compare treatment effects adjusting for covariates. Sensitivity analyses will be conducted to assess estimation biases.

## Supplementary information

Supplementary information

## Data Availability

The datasets generated and/or analyzed during the current study are not publicly available because the trial is ongoing and to protect patient privacy, but are available from the corresponding author on reasonable request after trial completion, through a data sharing agreement.
